# Impairment of enzymatic antioxidant defenses is associated with bilirubin-induced neuronal cell death in the cerebellum of Ugt1 KO mice

**DOI:** 10.1038/cddis.2015.113

**Published:** 2015-05-07

**Authors:** G Bortolussi, E Codarin, G Antoniali, C Vascotto, S Vodret, S Arena, L Cesaratto, A Scaloni, G Tell, A F Muro

**Affiliations:** 1 Mouse Molecular Genetics Group, International Centre for Genetic Engineering and Biotechnology, Trieste, Italy; 2 Department of Medical and Biological Sciences, University of Udine, Udine, Italy; 3 Proteomics and Mass Spectrometry Laboratory, ISPAAM, National Research Council, Naples, Italy

## Abstract

Severe hyperbilirubinemia is toxic during central nervous system development. Prolonged and uncontrolled high levels of unconjugated bilirubin lead to bilirubin-induced encephalopathy and eventually death by kernicterus. Despite extensive studies, the molecular and cellular mechanisms of bilirubin toxicity are still poorly defined. To fill this gap, we investigated the molecular processes underlying neuronal injury in a mouse model of severe neonatal jaundice, which develops hyperbilirubinemia as a consequence of a null mutation in the Ugt1 gene. These mutant mice show cerebellar abnormalities and hypoplasia, neuronal cell death and die shortly after birth because of bilirubin neurotoxicity. To identify protein changes associated with bilirubin-induced cell death, we performed proteomic analysis of cerebella from Ugt1 mutant and wild-type mice. Proteomic data pointed-out to oxidoreductase activities or antioxidant processes as important intracellular mechanisms altered during bilirubin-induced neurotoxicity. In particular, they revealed that down-representation of DJ-1, superoxide dismutase, peroxiredoxins 2 and 6 was associated with hyperbilirubinemia in the cerebellum of mutant mice. Interestingly, the reduction in protein levels seems to result from post-translational mechanisms because we did not detect significant quantitative differences in the corresponding mRNAs. We also observed an increase in neuro-specific enolase 2 both in the cerebellum and in the serum of mutant mice, supporting its potential use as a biomarker of bilirubin-induced neurological damage. In conclusion, our data show that different protective mechanisms fail to contrast oxidative burst in bilirubin-affected brain regions, ultimately leading to neurodegeneration.

Unconjugated bilirubin (UCB) is the metabolic product of heme degradation in mammals. The enzymes heme oxygenase 1 and 2 (HO-1, HO-2) catalyze the degradation of heme into biliverdin, which is subsequently reduced to bilirubin by biliverdin reductase. In the liver, UCB is conjugated to glucuronic acid by UDP-glucuronosil transferase (UGT1a1), rendering it water soluble and excretable in the bile. In neonates, the late induction of Ugt1a1 gene expression may result in a limited capacity to conjugate bilirubin. The imbalance between bilirubin production and its elimination result in neonatal hyperbilirubinemia. Moderate jaundice is present in a high proportion of babies and is considered beneficial because of the antioxidant and cytoprotective properties of UCB.^1^ However, excessive hyperbilirubinemia in newborns may produce acute bilirubin encephalopathy (BE), bilirubin-induced neurological disorders (BINDs) and eventually death by kernicterus.^2^ BIND is characterized by a wide array of neurological deficits, including irreversible abnormalities in motor, sensitive and cognitive functions, because of UCB accumulation in the cerebellum, hippocampus and basal ganglia. Infants affected by null mutations in the Ugt1 gene develop Crigler–Najjar syndrome type I (CNSI).^3^ CNSI patients present high UCB plasma levels and are particularly exposed to BE and BIND if untreated. Current therapy initially consists in intensive phototherapy (PT) but liver transplantation is required later in life because of a reduction in PT efficiency.^4, 5^

Despite extensive investigations in animal models and *in vitro* tissue culture cells, the basic mechanisms of hyperbilirubinemia neurotoxicity have not been fully clarified yet.^6, 7^ UCB affects a large number of cellular functions and neurological damage appears to be the result of their concerted disruption rather than misregulation of a single pathway. The reported observations range from energy metabolism,^8^ cell proliferation,^9^ DNA and protein synthesis,^10^ receptor functionality,^11^ to neurotransmitter uptake and release.^12^ Our group and others reported that UCB is associated with an increased oxidative stress condition in cell culture.^13, 14^ *In vitro* studies showed that exposure to UCB decreases neuronal and glia viability.^15, 16^ In primary cultures, it has been observed that UCB permeabilizes mitochondrial membranes, resulting in mitochondrial swelling, release of cytochrome *c* into the cytosol, caspase-3 activation, Bax translocation and cell death by apoptosis.^14, 17^ Bilirubin also decreases NGF signaling to AKT and ERKs, interfering with prosurvival signaling pathways.^18^ However, most of the studies leading to potential mechanisms of bilirubin neurotoxicity were performed on monotypic cultures with artificial dosing of bilirubin, lacking the complexity of the interactions between different cell types, as it occurs *in vivo*.

The aim of this study was to get a deeper unbiased insight into the molecular processes underlying bilirubin-induced neurodegeneration *in vivo* by using a mouse model of neonatal hyperbilirubinemia that shows early lethality because of bilirubin-induced neurological damage.^19^ Previous experiments conducted by our lab showed that the cerebellum of the Ugt1a^−/−^ mouse is the most vulnerable region of the brain to bilirubin toxicity.^19, 20^ Cerebellar susceptibility to bilirubin resulted in important alterations of its architecture, being the external germinal layer (EGL) and the Purkinje cell layer (PCL) the most affected regions, associated with an increase of terminal deoxynucleotidyl transferase-mediated deoxyuridine triphosphate nick end labeling (TUNEL)-positive cells.^19, 20^

In this study, we performed a differential proteomic analysis of the cerebellum from Ugt1 mutant and wild-type (WT) mice followed by validation of candidate genes both at protein and RNA levels. Our data point to an impairment of enzymatic antioxidant processes as important intracellular mechanisms involved in the onset of bilirubin-induced neurotoxicity *in vivo*.

## Results

### The mouse model

**Figure 1:**
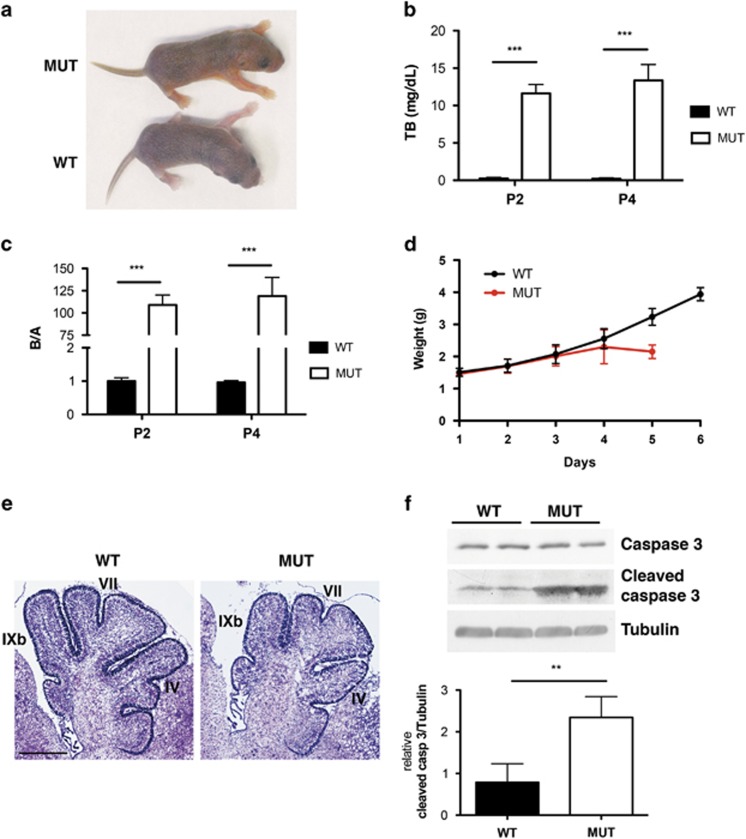
Ugt1 KO mutant mice phenotype and neurological damage. (**a**) As a consequence of Ugt1a inactivation, the mutant pup (top) appears jaundiced compared with its WT littermate (bottom). (**b**) Total plasma bilirubin levels were determined at post natal days 2 and 4 in WT and mutant mice (*n*≥4 time per genotype). Values represent the mean±S.D. (mg/dl). Two-way ANOVA, ***P<0.001. Interaction time genotype, NS; genotype, ***P<0.001; time, NS. (**c**) Bilirubin/albumin ratios were determined at P2 and P4 in WT and mutant mice (*n*≥4 time/genotype). Values represent the mean±S.D. of the calculated molar ratio between bilirubin and albumin. Two-way ANOVA, ***P<0.001. Interaction time genotype, NS; genotype, ***P<0.001; time, NS. (**d**) Weight curve of WT (black line, *n*=7) and mutant mice (red line, *n*=7). (**e**) Nissl staining of 4-day-old (P4) WT and mutant mice. IV, VII and IXb indicate the cerebellar fissures. Scale bar: 200 *μ*m. (**f**) Western blot analysis (top panels) of cerebellar protein extracts from P4 WT and untreated mutant mice, with anti-caspase-3 and anti-cleaved caspase-3 antibodies. Tubulin was used as loading control. Lower panel shows the densitometric quantification of WB analysis (*n*=4 genotype), results are expressed as the mean±S.D. of cleaved caspase-3/tubulin relative ratio

To get a deeper insight into the molecular processes underlying bilirubin-induced neuronal injury *in vivo*, we used a mouse strain bearing a targeted mutation in the Ugt1 gene.^19^ Mutant mice developed hyperbilirubinemia within 36 h after birth as evident by the yellow staining of their skin (Figure 1a, pup on the top). None of them survived up to 7 days after birth (50% survival was at P5).^20^ We monitored total bilirubin (TB) at postnatal day 2 (P2) and P4, showing that bilirubin levels rose rapidly after birth (Figure 1b) and were significantly different from WT littermates. As plasma albumin levels were unchanged between mutant and WT littermates, we calculated bilirubin/albumin (B/A) ratio. B/A ratio in mutant mice was 111- to 121-fold increased compared with WT littermates, at P2 and P4, respectively (Figure 1c). As hyperbilirubinemia proceeded, mutant mice showed severe neurological deficits and weight decrease. We daily monitored mutant mice weight since their birth and we noticed that the first signs of weight reduction preceded death by about 24 h (Figure 1d).

Nissl staining of brain sections showed that mutant mice had cerebellar hypoplasia and misshapen of cerebellar fissures IV, VII and IXb (Figure 1e), confirming prior results obtained with a less defined genetic background.^19^ We previously reported an increase in TUNEL-positive cells in the cerebellum of mutant mice,^19, 20^ indicating that bilirubin induces neuronal cell death. Western blot analysis of total cerebellar extracts from mutant mice at P4 showed 2.5-fold increased levels of cleaved caspase-3 (Figure 1f) compared with WT littermates.

### Differential proteomic analysis of cerebella from Ugt1 mutant and WT mice

As the cerebellum of mutant mice was severely affected by bilirubin toxicity, we applied a differential proteomic approach to reveal molecular effectors involved in bilirubin neurodegeneration and cell death *in vivo*. To reduce sample variability, we selected only male mutant animals showing weight loss at P4 and signs of severe neurological dysfunctions, such as lethargy and motor impairment, characterized by a marked improper posture of the rear limbs, poor and slow movement, and decreased feeding, as described previously.^19^ Animals showing these characteristics were expected to die the next day (P5). Cerebella from Ugt1 mutant and WT 4-d-old male mice (*n*=4 per genotype) were homogenized and total protein extracts were obtained. Corresponding SDS-PAGE profiles were comparable for all the analyzed samples (Supplementary Figure 1). The majority of protein species displayed a medium-low molecular weight without any trace of protein degradation, as evidenced by the presence of clear, sharp bands. These results confirmed the efficacy of the protein extraction protocol we used.

**Figure 2:**
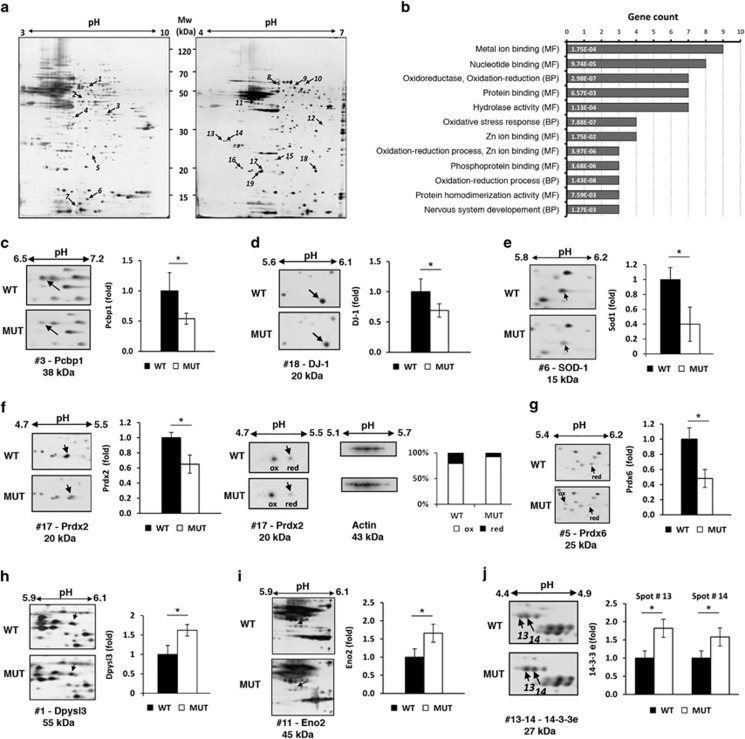
Changes in expression levels of selected proteins identified by proteomic analysis. (**a**) Representative proteomic maps of mutant mice cerebella. Mixture of cerebellar proteins were separated by 2-DE using two immobilized pH gradients, pH 3–10 (left) and pH 4–7 (right), 13-cm in length. Protein patterns were visualized by ammoniacal silver staining. Vertical and horizontal axes indicate apparent molecular mass and pI values, respectively. Differentially represented protein spots in the mutant mice are highlighted; numbering in Figure corresponds to numbering in Tables 1 and 2. These spots were subjected to nLC-ESI-LIT-MS/MS analysis for protein identification. (**b**) Functional clustering of the identified proteins. Functional enrichment analysis of the protein species identified by proteomic analysis according to molecular function (MF) and biological process (BP) is shown. GeneCodis analysis of the differentially represented proteins was performed. For simplicity, only the most representative functional categories are represented. The number of genes for each category is provided on horizontal axis and list only the first 13 co-occurrence terms. Statistical significance belonging to each category is shown within each bar. See Supplementary Table S2 in Supplementary Material for the full list of annotations. (**c–j**) Representative gel regions of 2-DE gels stained by ammoniacal silver were cropped. Protein variation in Ugt1 mutant mice (MUT) with respect to control (WT) are reported in the histograms. Histograms represent the normalized volume values of each spot of interests as obtained from four independent replicas. Data are the mean±S.D. of four independent experiments. Black boxes represent protein-normalized expression in the control (WT) mice cerebella, whereas white boxes represent the counterpart in the mutant mice cerebella. For Prdx2 (panel **f**), the histogram corresponding to the western blot analysis represents the relative amount of each forms (reduced or oxidized) expressed as percentage of the total amount of protein. Statistical significance is indicated by an asterisk, *P*<0.05

To analyze protein expression profile of cerebellar extracts, the samples for each genotype were pooled and subjected to two-dimensional electrophoresis (2-DE) analysis. About 200 and 150 reproducible spots were observed and quantified in the analyses performed in the range of pH 3–10 and 4–7, respectively (Figure 2a). As expected, the distribution of spots across the gel was not homogeneous, with a prevalence of focalized spots in the 20–70 kDa mass range and in the 4–7 pI range. These proteomic maps were also characterized by the presence of some horizontal spot trains with different pI values, which were associated with the same protein entry (see below), possibly as the results of post-translational modifications.

Nineteen protein spots (7 and 12 from the analyses in the range of pH 3–10 and pH 4–7, respectively), displayed a statistically significant change in abundance (Figure 2 and Supplementary Figure 2). The distribution of the differentially represented protein spots across the different replica gels was homogeneous, thus giving reliability to the analysis we performed.

Differentially represented proteins, plus those being invariant (used as reference spots), were then excised from the gels and analyzed by nanoLC-ESI-LIT-MS/MS for protein identification (Tables 1 and 2). Data searching in a non-redundant sequence database allowed the identification of 31 protein species having molecular masses ranging from 10 to 70 kDa. For 10 differentially represented spots, identification corresponded to a unique protein species; in the remaining ones (9 in number), two or more components were identified.

Tables 1 and 2 show the features of each spot, such as gel coordinates, protein coverage, relative mutant/WT protein abundance ratio and known functional properties. All identified proteins were classified in two groups according to the change direction in their relative abundance. Table 1 includes the protein species found to be down-represented in the cerebella of mutant mice compared with WT counterpart, whereas Table 2 lists the over-represented protein species.

Identified proteins were then analyzed for their functional properties in order to evaluate their association with common biological processes. This analysis was performed using GeneCodis software, which divides proteins into clusters according to the molecular function and the biological process (Figure 2b). The major functional groups contained proteins involved in oxidation reduction processes or with oxidoreductase activity (e.g., peroxiredoxin 2, Prdx2; peroxiredoxin 6, Prdx6; superoxide dismutase, Sod1), as well as proteins involved in nucleotide-binding or metal ion binding (e.g., guanine nucleotide-binding protein G(o) subunit α, Gnao; enolase 2, Eno2). Worth mentioning was the strong enrichment in enzymatic scavengers (Prdx2, Prdx6, DJ-1 and Sod1) and development of nervous system (e.g., NADH dehydrogenase [ubiquinone] flavoprotein 2, Nduv2) (Supplementary Table 2).

In order to validate 2-DE-based proteomic data and/or to discriminate between the multiple proteins identified within the same differentially represented spot, cerebella samples were also subjected to two-dimensional western blotting analyses with specific antibodies (Supplementary Figure 3). Validation was performed on an independent set of biological replicas by analyzing pooled samples from four additional mice per genotype. In addition, individual cerebella samples were also analyzed by one-dimensional western blot analysis (SDS-PAGE followed by WB), to rule out any bias generated by the use of pooled samples (Supplementary Figure 4). In all cases, western blotting results were in agreement with proteomic data, thus demonstrating a lower representation of spots of Pcbp1, Prdx2, Prdx6, Pak7/Dj-1 and Sod1 in the samples from Ugt1 mutant mice, and an increased representation therein of dihydropyrimidinase-like 3 (Dpysl3), 14-3-3e and Eno2.

Notably, western blotting of Prdx2 (Figure 2f) revealed that the protein abundance decrease, as measured by 2-DE in the mutant mice, was associated with the loss of the protein reduced form. It also demonstrated a concomitant increase of the oxidized, more acidic form of the protein, probably resulting as a consequence of Cys oxidation to sulfinic/sulfonic acid.^21^ On the other hand, a down-representation of the more basic protein spot corresponding to the reduced form of Prdx6 (Figure 2g) was apparent in the 2-DE of the mutant cerebella, which was confirmed by western blotting analysis (Supplementary Figure 3).

**Figure 3:**
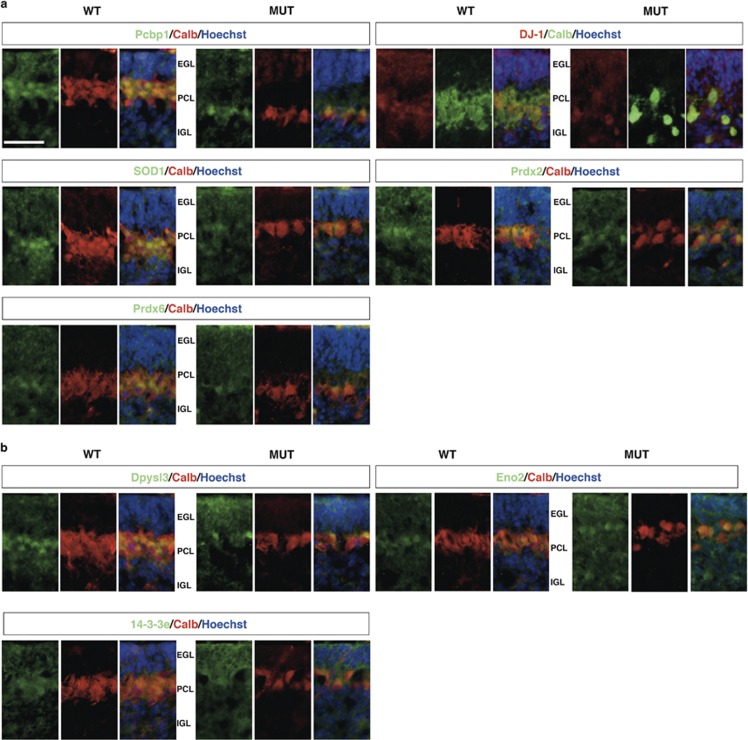
Immunohistochemical analysis of the candidate proteins. Representative fluorescent immunohistochemistry of cerebellar sections from WT and mutant mice using specific antibodies (green/red) and Hoechst (blue) to mark nuclei. (**a**) Down-represented proteins; (**b**) over-represented proteins. Calbindin antibody was used to detect PCs. Scale bar: 50 *μ*m. IGL, internal granular layer; EGL, external germinal layer; PCL, Purkinje cell layer

To highlight whether changes in the abundance of identified proteins were associated with specific cell types, we performed immunohistochemical analysis of the selected proteins. To improve spatial expression pattern discrimination, we colocalized those proteins with Purkinje cell (PC)-specific immunostaining (Figure 3). Among the down-represented proteins, we observed that Pcbp1, DJ-1, Sod1 and Prdx2 were mainly localized in the EGL and PCs layer, with poor staining in IGL (Figure 3a). In contrast, Prdx6 staining was more intense in the PCs layer and localized in the cytoplasmic compartment of PCs. Generally, the loss in protein content was associated with reduction in granule neurons (GNs) and PCs of the EGL and PCs, respectively.

In addition, immunostaining of the over-represented proteins (Figure 3b) revealed that Dpysl3, Eno2 and 14-3-3e were mainly localized in the PCL of the WT cerebellum, with poor staining in the EGL and IGL. On the contrary, we observed an increased staining of these proteins in the PCL and EGL of the mutant mice. As in the down-represented group, all proteins colocalized with PCs marker, although not all the calbindin-positive PCs expressed those proteins at equal levels.

### Changes in the protein levels are related to post-translational mechanisms

**Figure 4:**
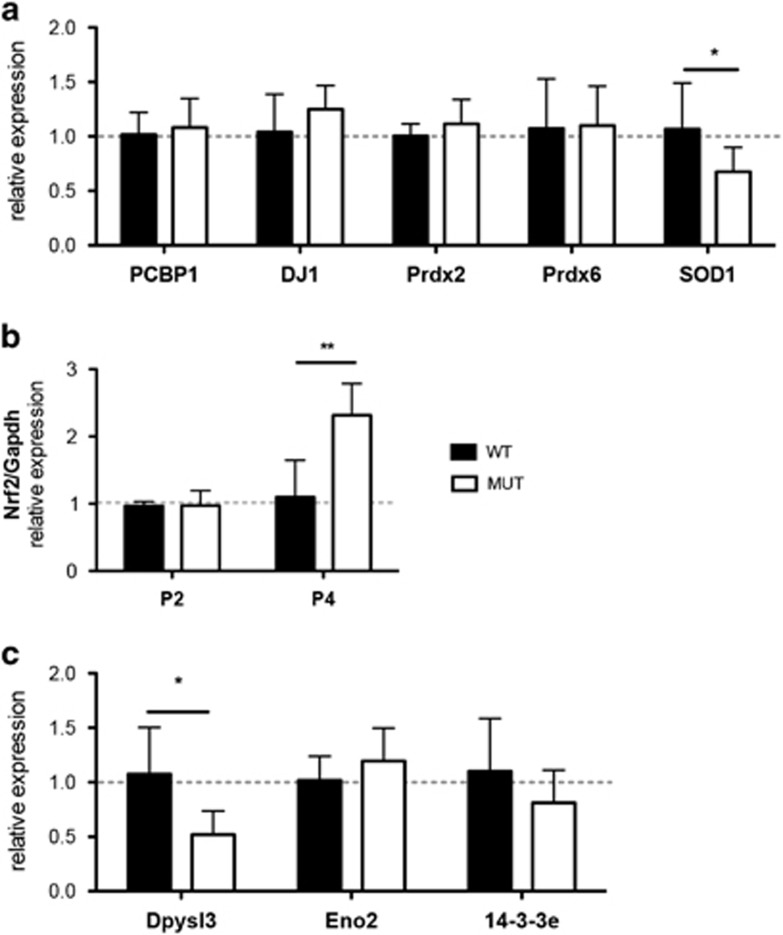
Relative expression of mRNA as assessed by qRT-PCR. (**a**) Candidate proteins down-represented in 2-DE, were validated at the mRNA level. WT levels were considered as one. Results are expressed as the mean±S.D. *t-*test *P<0.05. (**b**) Expression levels of NF-E2-related factor 2 (Nrf2) at postnatal days 2 and 4 in WT and MUT cerebellar extract. Two-way ANOVA, interaction time genotype *P<0.05; genotype *P<0.05; time **P<0.01. Values represent the mean±S.D. (**c**) mRNA expression levels of over-represented candidate proteins. Results are expressed as the mean±S.D. *t-*test *P<0.05

To determine whether the observed differences in protein levels were the consequence of significant changes in the steady state of their mRNAs, we quantified the corresponding mRNA levels at P4 by qRT-PCR. No significant differences in mRNA levels were observed for most of the analyzed genes (Figure 4a and c). The only exception among the group of under-represented proteins was Sod1, which showed a statistically significant decrease in mRNA levels (Figure 4a), as the protein one (Table 1,Figure 2e and Supplementary Figures 3D and 4). As NF-E2-related factor 2 (Nrf2) is a master cellular sensor of oxidative stress,^22^ we measured its corresponding mRNA levels by qRT-PCR. As shown in Figure 4b, Nrf2-mRNA levels were not affected at P2, whereas a significant upregulation was observed at P4, confirming the induction of an antioxidant response associated with bilirubin toxicity.

Among the group of over-represented proteins, despite the observed increase in protein levels (Table 2), Dpysl3 showed decreased mRNA levels, whereas no significant changes in Eno2 and 14-3-3e were observed between mutant and WT cerebellar RNA preparations (Figure 4c). All together, these results point to post-translational processing as the main mechanism regulating protein levels in the cerebellum of Ugt1^-/-^ mutant mice.

### Bilirubin-induced cell death is associated with increased Eno2 in the serum of the mutant mice

**Figure 5:**
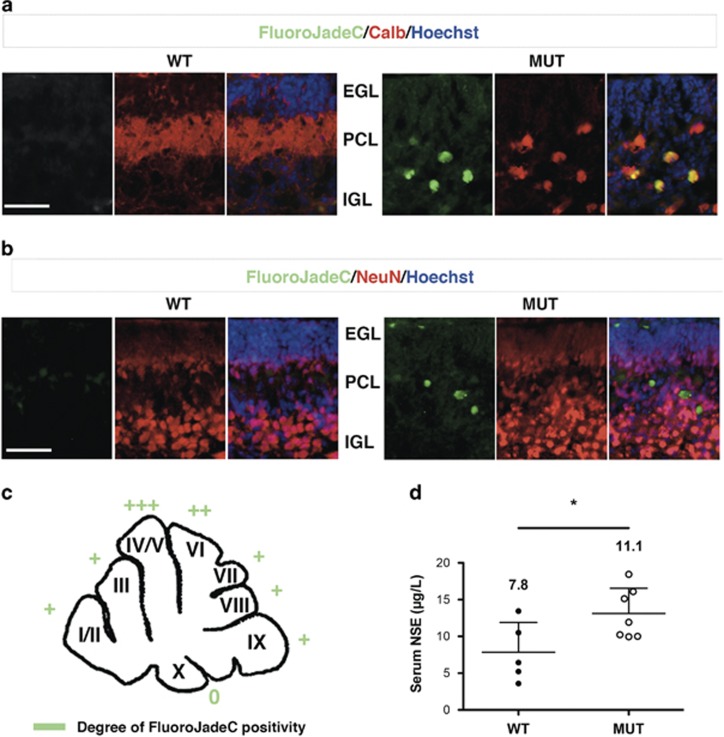
Assessment of bilirubin-induced cell death in the cerebellum at postnatal day 4. (**a**) Representative fluorescent immunohistochemistry of cerebellar sections from WT and mutant mice using FluoroJadeC (green) to detect degenerating neurons, colocalized with calbindin antibody (red) to highlight PCs. (**b**) FluoroJadeC staining was performed together with NeuN immunostaining (red) to highlight GNs. Hoechst (blue) was used to mark nuclei. Scale bar: 50 *μ*m. IGL, internal granular layer. (**c**) Cartoon reporting FluoroJadeC positivity along the entire cerebellum. Values from different sections belonging to the same animal were averaged. Represented values resulted from the average of four sections per mutant animal (four animals). 0, less than one positive cell/fissure analyzed; +, 1–3 cells; ++, 3–5; +++, 5–10. (**d**) Serum from WT (*n*=5) and MUT (*n*=7) mice was collected at P4 and Eno2 was quantified by ELISA test. Each dot represents a single animal. The mean value for each genotype is indicated (mean±S.D.). *t-*test *P<0.05

As Eno2 in is a known marker of neurological damage, we investigated in detail neuronal cell death in the cerebellum of mutant mice, highlighting the neuronal cell type that was more affected by bilirubin toxicity. We performed colocalization immunostaining experiments by using cell-specific markers of PCs (calbindin) or mature GNs (NeuN), in combination with FluoroJadeC staining (Figure 5a and b). FluoroJadeC staining detects all degenerating neurons, regardless of the specific insult(s) or mechanism(s) of cell death.^23^ We observed that FluoroJadeC positivity was strong and colocalized with calbindin-positive cells in cerebellar sections from mutant mice (Figure 5a). In contrast, no obvious FluoroJadeC/NeuN colocalization was observed with NeuN (Figure 5b). Poorly or no positive cells were detected in cerebellar section from WT littermates stained with FluoroJadeC. Figure 5c recapitulates FluoroJadeC positivity along the cerebellum of mutant mice. We observed that degenerating neurons were more abundant in the IV, V and VI fissures (average of 3–10 cells per section); whereas fissures I, II, VII, VIII and IX were less damaged (average of 1–3 cells per section). FluoroJadeC-positive signal in cerebellar fissure X was rarely observed.

Next, we evaluated the quantitative levels of Eno2 in the serum of the mutant and WT littermates by ELISA test. Eno2 has been extensively used as a marker of neuronal injury.^24, 25^ Indeed, we observed an increase of Eno2 serum levels in mutant mice (Figure 5d). In particular, values were 7.7 and 11.1 *μ*g/l for WT and mutant mice, respectively (*t*-test, *P<*0.05).

### P38 pathway is activated in the cerebellum of mutant mice

**Figure 6:**
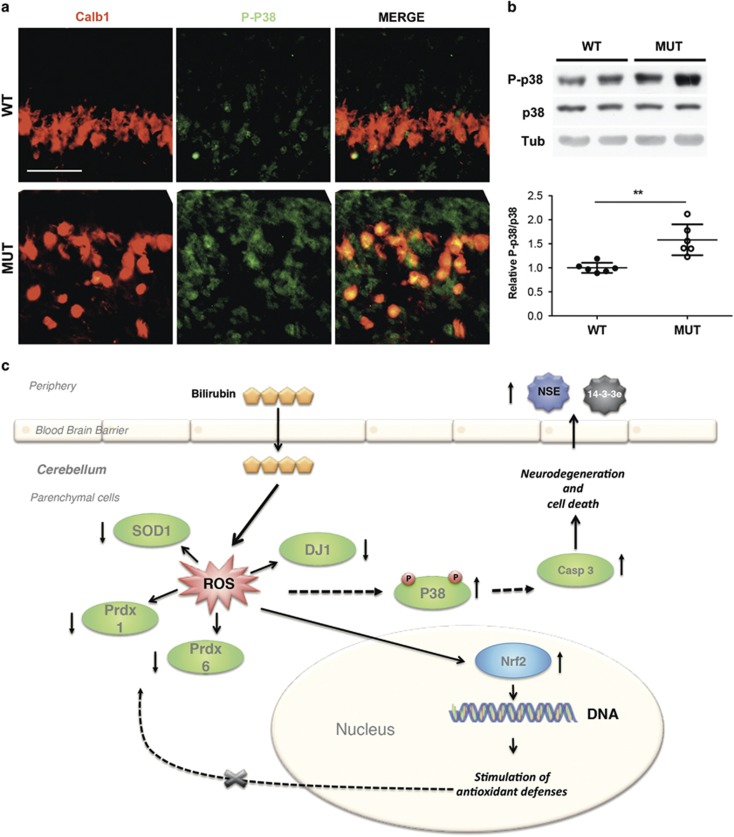
Cerebellar bilirubin-induced neurotoxicity in neonatal mice is associated with the activation of P38 pathway. (**a**) Representative fluorescent immunohistochemistry of cerebellar sections of WT and mutant mice using P-p38 antibody (green), anti-calbindin-1 antibody (red) to mark PCs and Hoechst (blue) to mark nuclei. Scale bar: 50 μm. IGL, internal granular layer. (**b**) Western blot analysis of total cerebellar protein extracts using anti-P-p38 and -p38 antibodies in WT and MUT mice. Tubulin was used as loading control. Lower panel shows the densitometric quantification of the bands. Error bars S.D. *t-*test **P<0.01. (**c**) Model of cerebellar bilirubin-induced neurotoxicity in neonatal mice. Severe hyperbilirubinemia *in vivo* leads to cerebellar neurodegeneration and neuronal cell death. Despite increasing transcription levels of Nrf2, a key sensor of oxidative stress that regulates gene expression of antioxidant genes, proteins involved in the cellular antioxidant defenses are down-represented in the cerebellum of mutant mice. Increase in p38 MAPK phosphorylation leads to apoptosis, as demonstrated by the activation of caspase-3 and the increase in TUNEL-positive cells.^19, 20^ As a consequence of neuronal cell death, neuronal-specific enolase (NSE) is released into the body fluids and then detected in the serum of Ugt1 mutant mice

Interestingly, six out of the eight selected proteins (Pcbp1, DJ-1, Sod1, 14-3-3e, Prdx2 and Prdx6) are connected to the mitogen-activated protein kinase p38 pathway.^26, 27, 28, 29, 30^ Therefore, to verify the involvement of p38 in bilirubin-mediated stress, we performed immunohistochemical and western blot analysis of cerebella from mutant and WT mice at P4, using p38 and phospho-p38-specific antibodies. As shown in Figure 6a, the phospho-p38 signal was increased in the cerebella of mutant mice. In the PCL, the signal colocalized with PC, as determined by PC-specific calbindin-1 staining. Phospho-p38 signal increase was also observed in the EGL. Western blot analysis showed a significant increase in phospho-p38 signal in the protein extracts from mutant mice cerebella (Figure 6b), indicating an activation of the p38-signaling pathway by bilirubin.

## Discussion

The mechanisms involved in bilirubin neurotoxicity to the developing nervous system are still poorly understood, and protein changes related to functional deficits are difficult to be established. We applied a proteomic approach to a mouse strain containing a null mutation of the Ugt1 gene with the aim of shedding light on the mechanisms and molecular effectors involved in bilirubin-associated neurotoxicity *in vivo*. Proteomic analysis demonstrated a lower representation of Pcbp1, DJ-1, reduced forms of Prdx2 and Prdx6, Sod1, Pfdn5, Tctp and Pebp1 in cerebella from Ugt1 mutant mice, and an increased representation therein of Dpysl2, Dpysl3, 14-3-3e, Vat1, Atp6v1a and Eno2.

### The mouse model

The mutant mice used in this study are characterized by severe neonatal hyperbilirubinemia leading to early death by bilirubin neurotoxicity.^19^ They display many of the clinical and histological symptoms of BIND, including severe motor impairment, poor and slow movement, decreased feeding, and major changes in cerebellar architecture. The original strain of mutant mice displays an important variability on the onset of the disease symptoms and death, ranging from P4 to P11.^19^ To reduce sample variability increasing the powerfulness of the proteomic approach, we adopted the following strategy: (a) we increased the homogeneity in the genetic background by using animals after >9 backcrosses to WT C57Bl/6 mice (with at least 99.8% of C57Bl/6 genetic background), which resulted in a sharper mortality curve (death of mice ranging from P4 to P7, with 50% survival at P5); (b) we daily monitored the weight of pups because a drop in the weight curve indicated a critical condition that resulted in animal death after 24 h; and (c) we analyzed at P4 only those pups having a weight drop. These aspects resulted in higher homogeneity of the samples, as evidenced in the 2-DE replica maps.

### Oxidative stress

The proteomic analysis performed in 4-day-old mice cerebellar specimens allowed the identification of protein changes associated with early bilirubin-induced neuronal damage. This comparative analysis revealed that the most affected biological process was the cellular response to oxidative stress. Oxidative stress is considered a major mechanism of bilirubin-induced cytotoxicity, as demonstrated by *in vitro* studies,^15, 16, 31, 32^ although confirmation of this hypothesis still needs *in vivo* experimental data. In fact, studies performed on Gunn rat pups showed that lipid peroxidation is not the main mechanism of BIND,^33^ and there are no reports yet addressing protein oxidation as a mechanism related to bilirubin cytotoxicity *in vivo*. However, in some diseases states in which free radical production have a main pathogenic role, such as Alzheimer's disease, biliverdin reductase undergoes post-translational oxidative modifications, which may reduce local bilirubin production thus affecting the overall antioxidant capacity.^34^ Interestingly, mildly elevated bilirubin concentrations are considered antioxidant and, therefore, may assist in neutralizing reactive oxygen species (ROS) and preventing oxidative damage.^35^ A redox bilirubin–biliverdin cycling mechanism has been proposed to explain the antioxidant properties of bilirubin ^36, 37^ but this hypothesis was not further confirmed by others.^38, 39^

We also identified Prdx2 and Prdx6, members of the peroxiredoxin family of enzymes, which serve as scavengers of cellular peroxides and provide protection against oxidative stress.^40^ In the CNS, Prdx2 is almost selectively expressed in large neurons, such as hippocampal pyramidal and Purkinje neurons. Hence, oxidation of these proteins could lead to their increased susceptibility to proteosomal degradation and a decrease in their protein levels. Indeed, protein oxidation leads to alterations in protein expression and gene regulation, protein turnover, modulation of cell signaling and induction of apoptosis or necrosis.^41^ Concomitantly, the Nrf2 transcription factor, a central factor in the adaptive cell response to oxidative stress,^22^ was upregulated, confirming a central role of the oxidative stress in bilirubin toxicity. However, *in vivo*, Nrf2 increase was not sufficient to activate its target genes involved in ROS stress response, as evidenced by the absence of mRNA levels stimulation and the decreased protein amounts, probably because of the acute and rapid increase in bilirubin and consequent irreversible cell damage (Figures 4b and 6).

The integral mitochondrial protein DJ-1 is associated with oxidative stress and scavenges H_2_O_2_.^42^ It behaves as a cellular redox sensor and, under oxidative stress conditions or proteasome block, it exerts its neuroprotective effects preventing aggregate formation.^43^ Moreover, DJ-1 protects cells from apoptotic cell death triggered by ROS, and modulates transcription of antioxidant genes.^22^ An involvement of DJ-1 in UCB-induced damage was already reported by our group and others.^13, 44^ In fact, increased levels of DJ-1 in primary cultures of hippocampal neurons were reported after 4 h of bilirubin induction, and in SH-SY5Y cells after 24 h of bilirubin treatment.^44, 45^ However, in this study we observed a decrease in DJ-1 levels in the cerebella of mutant mice chronically affected by hyperbilirubinemia. Our observation is in line with previous results obtained in primary hippocampal neuron cultures treated with bilirubin, where DJ-1 levels decreased 24 h post-treatment.^44^ Thus, the observed increase in cell death in the cerebella of the hyperbilirubinemic pups may be related to the decrease in DJ-1 levels, as detected in the present work.

Another primary cellular defense to oxidative insults is the scavenger enzyme Sod1. This enzyme catalyzes the rapid conversion of superoxide anion into molecular oxygen and hydrogen peroxide. Sod1 mutations are associated with familiar amyotrophic lateral sclerosis.^30^ Indeed, Sod1 deletion results in increased oxidative stress and axonal degeneration *in vivo*.^46^ Interestingly, we provided the first evidence that Sod1 is down-represented at the protein level in association with hyperbilirubinemia in the central nervous system (CNS). Our observation is in line with the observation that Sod1 is downregulated at the mRNA level in the myocardium of the Gunn rat.^47^

Dpysl3, a member of the TUC (TOAD-64/Ulip/CRMP) family, known also as CRMP-4 or TUC-4, is involved in neuronal plasticity and neurite outgrowth and extension.^48^ Our results are in line with the observed increase in Dpysl3 when SH-SY5Y neuronal cells were treated with UCB.^45^ However, further experiments are needed to understand the observed differences between Dpysl3 mRNA and protein levels.

Pcbp1 is an RNA-binding protein that interacts in a sequence-specific manner with single-stranded poly(C). It performs multiple functions including mRNA stabilization,^49^ translational silencing ^50^ and translational enhancement.^51^ Although Pcbp1 is known to affect the stability of gene expression and axon maturation through post-transcriptional regulation, its exact functions in neurons are still unclear and no report so far describes its possible relation with bilirubin-induced cytotoxicity. However, PCBP1 depletion in human SH-SY5Y cells affected a number of transcripts belonging to different categories with pathway involvement in Wnt (WNT2B, WNT4 and WNT7B) signaling, TGF*β* signaling, translation factors and nuclear receptors.^52^

We have also noticed that six out of the eight selected proteins (Pcbp1, DJ-1, Sod1, 14-3-3e, Prdx2 and Prdx6) were reported to be connected to the mitogen-activated protein kinase p38 pathway,^26, 27, 28, 29, 30^ which mediates a wide variety of cellular behaviors ranging from response to stress stimuli and cell cycle. In particular, p38 activation occurs in response to inflammatory cytokines, growth factors or other extracellular stimuli. Importantly, p38 acts both up- and downstream the apoptotic cascade. The downregulation of these proteins in the cerebella of mutant mice, as a consequence of increase in bilirubin, in both systemic and tissue levels,^19, 53^ and ROS, results in p38 activation, apoptosis and release of neuronal markers to the body fluids (Figure 6b). The results presented here confirm previous *in vitro* studies on cerebellar GNs and cortical astrocytes, where p38 was activated in response to bilirubin.^54, 55^ Interestingly, the P-p38-positive signals colocalized with the most affected cerebellar neurons (PCs and GNs of EGL). However, only PCs showed increased FluoroJadeC positivity, proving their degeneration and death. In contrast, GNs reduction was not associated with evident FluoroJadeC positivity; this, is probably linked to a delay in their replication cycle in the outer EGL caused by bilirubin rather than cell death (GB and AFM, unpublished data). Further studies will demonstrate whether the decrease in antioxidant defenses is associated with cell cycle regulation in the EGL of the mutant mice.

The cerebellar postnatal development of mice from birth to P7/P8 mirrors the window between 24 and 32 gestational weeks in humans.^20, 56^ This period coincides with the greatest vulnerability of babies to brain injury,^57^ leading to disturbance of myelination^58^ and neuronal growth. Indeed, the findings presented here may improve the understanding of the mechanisms associated to bilirubin neurotoxicity preterm babies.

### Biomarkers of neurological damage

Proteomics analysis is an important tool to detect reliable biomarkers exploitable for early detection of pathological states. 14-3-3 Proteins are a highly conserved group of molecules having pivotal roles in apoptosis, intracellular trafficking, cell cycle control and signal transduction, which modulate the interaction between components of signal transduction pathways.^59^ They are abundant in neurons (up to 1% of the total soluble protein in the brain), and were found in the cerebrospinal fluid of patients with cerebellar disorders,^60^ suggesting their possible use as biomarker of neuronal damage. Specific 14-3-3 isoforms are increased in several brain regions of aged patients with Alzheimer's disease and Down syndrome,^61^ similarly to what observed here in the case of cerebella of Ugt1 mutant mice.

In this study, we showed that the over-representation of Eno2 in the cerebellum of mutant mice correlates with a significant increase in its serum levels. However, the possible use of this neuro-specific protein as a marker of BIND is controversial. Increased in Eno2 levels in plasma and cerebrospinal fluid have previously been observed in Gunn rats.^62^ In contrast, piglets infused with bilirubin showed no differences of Eno2 levels in serum and cerebrospinal fluid, despite significant changes in auditory brainstem response.^63^ Studies on jaundiced neonates (serum bilirubin ≥ 20 mg/dl) did not show any correlation between the serum Eno2 and bilirubin values.^64^ Conversely, infants having auditory neuropathy presented significantly higher Eno2 levels.^64^ Our data on severely affected hyperbilirubinemic mice support Eno2 as a reliable biomarker of BIND, but its possible use in neonates still needs further studies.

In conclusion, we show here that severe hyperbilirubinemia *in vivo* leads to cerebellar neurodegeneration and neuronal cell death. By using a 2-DE-based proteomic approach, we demonstrated that several proteins involved in the cellular antioxidant defenses are down-represented in the cerebellum of the Ugt1^−/−^ mouse model. Protein level changes were mainly the consequence of post-translational mechanisms because corresponding mRNAs did not show concomitant variations with respect to control. In addition, we here provide evidence that neuronal-specific Eno2 (found to be over-represented in the cerebellum of hyperbilirubinemic mice) was increased in the plasma of these animals. The information resulting from this study provides new insights into the molecular mechanisms associated with bilirubin-induced neurotoxicity *in vivo*, suggesting that an improvement of antioxidant defenses may be of crucial importance to prevent cerebellar neurodegeneration.

## Materials and Methods

### Animal model

Ugt1 mutant mice have been described previously.^19, 20, 53^ WT littermates were used as a control. Mice were housed and handled according to institutional guidelines, and experimental procedures approved by the International Centre for Genetic Engineering and Biotechnology (ICGEB) board. Animals used in this study were of 99% C57Bl/6 genetic background, obtained after >9 backcrosses with C57Bl/6 mice. Homozygous mutant animals were obtained by breeding heterozygous mating pairs. Animals were kept in a temperature-controlled environment with a 12–12 h light–dark cycle. They received a standard chow diet and water *ad libitum*. Four days after birth (P4), mutant mice and their WT littermates were killed. Brains were removed from the skulls and cerebella were divided in two hemispheres; one was subjected to protein extraction, whereas the other was subjected either to RNA extraction and/or brain histology.

### Bilirubin determination in plasma

Blood from P4 mice was collected as previously described.^19^ Total plasma bilirubin levels were determined with the Direct and Total Bilirubin Reagent Kit (BQ Kits, Inc., San Diego, CA, USA) as previously described.^20^

### Albumin determination

Albumin determination in plasma samples was performed with the Bromocresol Green method, adapting the method to use minimal volumes (2 *μ*l) as previously described.^53^ For each test, a standard curve was performed by diluting of a stock solution (10 mg/ml) of human albumin (Albuman; 200 g/l, Sanquin, Amsterdam, The Netherlands) in water. Absorbance values at 630 nm were obtained by using a multiplate reader (Perkin Elmer Envision Plate Reader, Walthman, MA, USA). Results were expressed as g/l.

### Preparation of mouse cerebellar proteins

Total protein extracts were prepared as already described,^65^ with minor modifications. Each cerebellum (about 30 mg of tissue) was homogenized using a Potter Dual supplied with a PTFE pestle and mechanical agitator (ForLab, Bergamo, Italy) in a solution (3 ml) containing 7 M urea, 2 M thiourea, 4% w/v CHAPS, 0.3% w/v dithiothreitol, IPG buffer (0.5% v/v ampholytes), Protease Inhibitor Cocktail (Sigma, Milan, Italy) and a trace of bromophenol blue dye, until >90% of the tissue was lysed. Disrupted tissues were then sonicated and homogenates were centrifuged at 21 000 x *g* for 30 min to separate non-solubilized sediment material. A portion of each homogenate was pooled to obtain a mixture of mutant and WT mice cerebellar proteins, respectively. These samples were further subjected to proteomics analysis. Four biological replicas for each genotype were used for proteomic analyses.

### Two-dimensional polyacrylamide gel electrophoresis

Fifty or eighty micrograms of protein extracts were loaded onto 13 cm, pH 3–10 L or pH 4–7 L IPG strips, respectively (Amersham Biosciences, Milan, Italy). Performing 2-DE with two pI ranges allowed a better quantification of protein components not properly resolved using a single experimental condition. To evaluate technical reproducibility, gel analyses were performed in technical quadruplicate and quantified for statistical variation. IEF was conducted using an IPGPhor II system (Amersham Biosciences) according to the manufacturer's instructions. Focused strips were equilibrated as described previously ^66^ and applied directly to 12% SDS-polyacrylamide gels and separated at 130 V. Gels were fixed and stained by ammoniacal silver. For each pool, four experimental replicates were subjected to 2-DE. All gels images were acquired with an Image Scanner II apparatus (GE Healthcare, Milan, Italy).

### Evaluation of differentially represented spots

Gel images were analyzed by the Image Master 2D Platinum software (GE Healthcare) that allowed performing a comparative image analysis, as previously described.^66, 67^ Protein spots were detected and matched between the different samples; individual spot volume values were obtained according to the program instruction and normalized using the program volume normalization function. The ratio for the candidate protein spots was compared with each other and a mean relative difference in spot intensity was calculated. Differences in protein spot expression levels were considered as statistically significant when *t*-test resulted in a *P*-value<0.05.

### Mass spectrometry analysis

Identification of the selected spots was performed as previously described.^68^ Briefly, spots from 2-DE were manually excised from gels, minced and washed with water. Proteins were *in-gel* reduced, S-alkylated and digested with trypsin. Tryptic digests were desalted and analyzed by nLC-ESI-LIT-MS/MS using a LTQ XL mass spectrometer (Thermo, San Jose, CA, USA) equipped with a Proxeon nanospray source connected to an Easy-nanoLC (Proxeon, Odense, Denmark).^69^ Peptide mixtures were separated on an Easy C_18_ column. Spectra acquisition (in the range *m/z* 400–2000) was controlled by a data-dependent product ion scanning procedure over the three most abundant ions, enabling dynamic exclusion (repeat count 2 and exclusion duration 60 s); the mass isolation window and collision energy were set to *m/z* 3% and 35%, respectively.

Raw data files from nLC-ESI-LIT-MS/MS experiments were searched with MASCOT software package version 2.2.06 (Matrix Science, London, UK) against an updated Mammalia non-redundant sequence database (UniProt 2010/12). Database searching was performed by selecting Cys carbamidomethylation as a fixed modification, whereas Met oxidation and pyroglutamate formation at N-terminal Gln were considered as variable modifications, respectively. It was also carried out by using a mass tolerance value of 2 Da for precursor ion and 0.8 Da for MS/MS fragments, trypsin as proteolytic enzyme, and a missed cleavages maximum value of 2. Candidates with >2 assigned peptides with an individual MASCOT score >25, both corresponding to *P*<0.05 for a significant identification, were considered as confidently identified. Definitive peptide assignment was always associated with manual spectral visualization and verification. Protein identification was checked manually and further evaluated by comparison with their calculated mass and pI values, using the experimental values obtained from 2-DE.

### Gene annotations co-occurrence analysis

Gene IDs corresponding of differentially represented proteins identified by proteomics analysis were submitted to GeneCodis (http://genecodis.cnb.csic.es/), a web-based tool for ontological analysis, selecting *Mus musculus* as the source for the annotations and two gene ontology categories (molecular function and biological process) to perform the gene annotation co-occurrence analysis.

### Western blot analysis

To validate proteomic data, protein extracts from an independent set of biological replica made of pooled samples from four Ugt1 mutant and four WT mice were loaded onto 7 cm IPG strips, separated by IEF and SDS-PAGE and then transferred overnight to nitrocellulose support (Schleicher & Schuell, Keene, NH, USA). Membranes were saturated by incubation with 5% w/v non-fat dry milk in PBS–0.1% w/v Tween 20 at 4 °C, overnight. Then, blots were incubated with the primary antibody (listed in Supplementary Table 3) at 4 °C, overnight, washed and then incubated with the secondary antibody coupled to peroxidase (Sigma, St. Louis, MO, USA) for 60 min. Blots were developed using the enhanced chemiluminescence procedure (PIERCE, Rockford, IL, USA). Actin or tubulin were used as loading controls. Blots were quantified by using a Chemi DOC XRS densitometer (Bio-Rad, Hercules, CA, USA). To rule out any bias generated by the use of pooled samples, western blotting was also performed on individual cerebella resolved by SDS-PAGE.

### Preparation of total RNA from the mouse cerebellum and real time PCR analysis

Total RNA from mouse cerebellum was prepared using EuroGOLD Trifast (Euroclone, Milano, Italy), according to the manufacturer's instructions. One microgram of total RNA was reverse-transcribed using M-MLV (Invitrogen, Carlsbad, CA, USA) and oligo-dT primers according to the manufacturer's instructions. Total cDNA (1 *μ*l) was used to perform qPCR using the specific primers listed in Supplementary Table 1. qPCR was performed using the iQ SYBR Green Supermix (Bio-Rad) and a C1000 Thermal Cycler CFX96 Real Time System (Bio-Rad). Expression of the gene of interest was normalized to the Gapdh house-keeping gene. Data were analyzed using the ΔΔCt method.

### Brain histology and immunofluorescence

Total brains from each genotype were extracted and fixed with 4% PFA in PBS at 4 °C, overnight. After cryoprotection in 20% sucrose, 0.02% sodium azide in PBS, specimens were frozen in cryostat embedding medium (Bio-optica, Milano, Italy) and 14 *μ*m sagittal sections were obtained in a cryostat. Nissl staining was performed as previously described.^19^ For immunofluorescence, 14 *μ*m sagittal sections were blocked in PBS containing 2.5% BSA and 0.3% Triton X-100, for 2 h, at room temperature (RT). After blocking, specimens were incubated with the primary antibodies listed in Supplementary Table 3 in blocking solution, for 2 h, at RT. After three washes with blocking solution for 5 min, specimens were incubated with the secondary antibody (Alexa Fluor 488 or 568, Invitrogen) for 2 h, at RT. FluoroJade C (Millipore, Darmstadt, Germany) was used to visualize all degenerating neurons. Briefly, slides were treated with 1% NaOH in 80% ethanol for 5 min and then rinsed for 2 min in 70% ethanol, for 2 min in PBS and then incubated in 0.06% potassium permanganate (Sigma-Aldrich, Milano, Italy) solution for 5 min. After three washes with PBS, slides were subjected to immunofluorescence procedure as described above. Following secondary antibody washings, slides were then transferred to a 0.0001% solution of FluoroJade C dissolved in 0.1% acetic acid, for 20 min. The proper dilution was accomplished by first making a 0.01% stock solution of the dye in distilled water according to the manufacturer's instructions. Working solution was prepared immediately before use. Nuclei were visualized by addition of Hoechst (10 μg/ml, Invitrogen) for 5 min after secondary antibody solution. Nissl-stained slides were mounted in Eukitt (Fluka, St. Louis, MO, USA), whereas immunostained slides were mounted in Mowiol 4–88 (Sigma Aldrich, Milano, Italy). Images were acquired using a Nikon Eclipse E-800 epi-fluorescent microscope bearing a charged-coupled device camera (DMX 1200F; Nikon, Amstelveen, The Netherlands). Digital images were collected using ACT-1 (Nikon) software.

### Quantification of serum neuronal-specific Eno2

Neuronal-specific Eno2 (NSE) was detected in serum samples of mutant and WT littermates at postnatal day 4 using the CanAg NSE EIA kit (Fujirebio Diagnostic, Inc., Göteborg, Sweden), according to the manufacturer's instructions.

## Figures and Tables

**Table 1:** List of the down-represented proteins in the cerebellum of Ugt1 mutant mice, as detected by 2-DE and identified by nanoLC-ESI-LIT-MS/MS analysis

**Spot #**	**Protein identity**	**SwissProt entry**	**Peptides**	**Unique peptides**	**MASCOT score**	**EmPAI score**	**Sequence coverage (%)**	**Mw kDa**	**pI**	**MUT/WT ratio**	**Function**
	*Poly(rC)-binding protein (Pcbp1)*	P60335	16	12	661	1.82	51.4	37.5 (38)	6.66 (6.7)	0.54	Single-stranded nucleic acid binding protein that binds preferentially to oligo dC
*3*	*Alcohol dehydrogenase 5 (class III), chi polypeptide (Adh5)*	Q6P5I3	6	6	336	0.31	17.9	39.6 (38)	6.97 (6.7)	0.54	S-(hydroxymethyl)glutathione dehydrogenase activity and alcohol dehydrogenase (NAD) activity
	*Acetyl-CoA acetyltransferase (cytosolic) (Acat2)*	Q8CAY6	4	4	184	0.29	18.1	41.3 (38)	7.16 (6.7)	0.54	Catalysis of the reaction: 2 acetyl-CoA = CoA + acetoacetyl-CoA
*4*	*Isocitrate dehydrogenase [NAD] subunit α, mitochondrial (Idh3a)*	Q9D6R2	14	10	400	0.71	27.9	36.7 (37)	6.27 (5.5)	0.56	NAD binding, isocitrate dehydrogenase (NAD+) activity and magnesium ion binding
	*Guanine nucleotide-binding protein G(o) subunit α (Gnao)*	P18872	6	6	325	0.70	18.4	40 (37)	5.34 (5.5)	0.56	Modulators or transducers in various transmembrane signaling systems
5	*Peroxiredoxin-6 (Prdx6)*	P30041	3	2	88	0.15	8.5	25 (25)	6.00 (6.0)	0.48	Involved in redox regulation of the cell, may have a role in protection against oxidative injury
*6*	*Superoxide dismutase [Cu-Zn] (Sod1)*	P08228	8	8	379	1.95	44.8	15.8 (13)	6.02 (5.9)	0.48	Destroys radicals that are normally produced within the cells and that are toxic to biological systems
*7*	*Putative uncharacterized protein (Pfdn5)*	Q6ZWY7	6	6	416	1.18	32.5	18 (10)	5.95 (5.6)	0.65	Interacts selectively and non-covalently with an unfolded protein
*16*	*Translationally controlled tumor protein (Tctp)*	P63028	9	6	368	2.52	33.1	19.5 (20)	4.76 (4.8)	0.69	Involved in calcium binding and microtubule stabilization
*17*	*Peroxiredoxin-2 (Prdx2)*	Q61171	9	6	434	2.61	31.8	21.8 (20)	5.20 (5.1)	0.65	Involved in redox regulation of the cell, may have an important role in eliminating peroxides generated during metabolism
*18*	*Parkinson disease (autosomal recessive, early onset) 7 (DJ-1)*	B2KFH8	13	8	475	3.06	54.8	19.9 (20)	6.32 (6.1)	0.69	Protects cells against oxidative stress and may act as an atypical peroxiredoxin-like peroxidase that scavenges H_2_O_2_. May function as a redox-sensitive chaperone
*19*	*Phosphatidylethanolamine-binding protein 1 (Pebp1)*	Q3TGC5	10	9	557	3.50	69.0	20.9 (19)	5.36 (5.0)	0.58	Putative uncharacterized protein

Spot number, protein description, accession number (SwissProt entry), theoretical and experimental (in parentheses) molecular mass and pI values, total and unique peptides detected, sequence coverage (%), MASCOT and EMPAI score values, fold increase with respect to control, and the known function of proteins are listed

**Table 2:** List of the over-represented proteins in the cerebellum of Ugt1 mutant mice, as detected by 2-DE and identified by nanoLC-ESI-LIT-MS/MS analysis

**Spot #**	**Protein identity**	**SwissProt entry**	**Peptides**	**Unique peptides**	**MASCOT score**	**EmPAI score**	**Sequence coverage (%)**	**Mw kDa**	**pI**	**MUT/WT ratio**	**Function**
	*Dihydropyrimidinase-related protein 3 (Dpysl3)*	Q3TT92	22	19	1081	1.39	48.2	62 (55)	6.04 (6.0)	1.72	Catalysis of the hydrolysis of any non-peptide carbon-nitrogen bond in a cyclic amide
*1*	*Seryl-aminoacyl-tRNA synthetase (Sars)*	Q8C483	13	11	522	0.42	25.6	61.2 (55)	7.13 (6.0)	1.72	Catalysis of the reaction: ATP + L-serine + tRNA(Ser) = AMP + diphosphate + L-seryl-tRNA(Ser)
*2*	*Putative uncharacterized protein (Vat1)*	Q3TXD3	4	4	198	0.18	17.5	37.5 (47)	6.66 (5.6)	2.32	Catalysis of a redox reaction; interacts selectively and non-covalently with zinc ions
*8*	*V-type proton ATPase catalytic subunit A (Atp6v1a)*	P50516	37	25	1536	3.15	45.7	68.3 (58)	5.42 (5.3)	1.72	Catalytic subunit of the peripheral V1 complex of vacuolar ATPase; acidifies a variety of intracellular compartments in eukaryotic cells
*9*	*Dihydrolipoyllysine-residue acetyltransferase component of pyruvate dehydrogenase complex, mitochondrial (Dlat)*	Q8BMF4	20	14	788	0.7	25.7	68.0 (56)	8.81 (5.6)	2.00	The pyruvate dehydrogenase complex catalyzes the overall conversion of pyruvate to acetyl-CoA and CO_2_
	*Dihydropyrimidinase-related protein 3 (Dpysl3)*	Q3TT92	7	6	380	0.42	16.0	61.8 (56)	6.04 (5.6)	2.00	It is involved in nervous system development having hydrolase activity and acting on carbon–nitrogen bonds, in cyclic amides
*10*	*Dihydropyrimidinase-related protein 2 (Dpysl2)*	O08553	18	13	866	1.24	31.1	62.3 (58)	5.95 (5.8)	1.56	Necessary for signaling by class 3 semaphorins and cytoskeleton remodeling, axon guidance, neuronal growth and cell migration
	*26S protease regulatory subunit 6B (Psmc4)*	Q3TUN5	23	19	1089	1.89	54.1	47.4 (45)	5.09 (5.1)	1.66	It has ATP binding and nucleoside-triphosphatase activity
*11*	*26S protease regulatory subunit 6A (Psmc3)*	Q3THI5	18	15	788	1.79	45.8	45.2 (45)	5.39 (5.1)	1.66	It has ATP binding and nucleoside-triphosphatase activity
	*Enolase (Eno2)*	Q3UJ20	13	11	669	0.83	32.5	47.3 (45)	4.99 (5.1)	1.66	Belongs to the enolase family
*12*	*Transaldolase (Taldo)*	Q93092	20	16	762	1.60	40.1	37.4 (33)	6.57 (6.2)	1.45	It is important for the balance of metabolites in the pentose-phosphate pathway
	*LIM and SH3 domain protein 1 (Lasp1)*	Q61792	9	8	419	1.09	34.2	29.9 (33)	6.61 (6.2)	1.45	Has an important role in the regulation of dynamic actin-based, cytoskeletal activities
*13*	*14-3-3 Protein epsilon (14-3-3e)*	P62259	39	22	1405	32.90	72.2	29.2 (27)	4.63 (4.5)	1.82	Adapter protein implicated in the regulation of both general and specialized signaling pathways
	*Tyrosine 3-monooxygenase/tryptophan 5-monooxygenase activation protein, beta polypeptide (Ywhab)*	A2A5N2	20	12	771	4.85	47.2	28.1 (27)	4.77 (4.5)	1.82	It has monooxygenase activity and protein domain-specific binding
*14*	*14-3-3 Protein epsilon (14-3-3e)*	P62259	36	19	1268	29.02	66.7	29.2 (27)	4.63 (4.6)	1.58	Adapter protein implicated in the regulation of both general and specialized signaling pathways
	*Tropomyosin alpha-4 chain (Tpm4)*	Q6IRU2	14	13	741	1.71	52.4	28.5 (27)	4.65 (4.6)	1.58	Binds to actin filaments in muscle and non-muscle cells, having a central role, with the troponin complex, in the calcium-dependent regulation of striated muscle contraction
*15*	*NADH dehydrogenase [ubiquinone] flavoprotein 2, mitochondrial (Nduv2)*	Q9D6J6	12	10	647	1.80	48.8	27.3 (23)	7.0 (5.3)	1.40	Core subunit of the mitochondrial membrane respiratory chain NADH dehydrogenase (complex I)
	*Apolipoprotein A-I (ApoA1)*	Q3V2G1	11	8	457	1.26	28.8	30.7 (23)	5.64 (5.3)	1.40	It has a role in lipid transport and binding

Spot number, protein description, accession number (SwissProt entry), theoretical and experimental (in parentheses) molecular mass and pI values, total and unique peptides detected, sequence coverage (%), MASCOT and EMPAI score values, fold increase with respect to control, and the known function of proteins are listed

## Abbreviations

UCB: unconjugated bilirubin
HO-1 and HO-2: heme oxygenase 1 and 2, respectively
UGT1a1: UDP-glucuronosil transferase
BE: bilirubin encephalopathy
BIND: bilirubin-induced neurological disorders
CNS: central nervous system
CNSI: Crigler–Najjar syndrome type I
PT: phototherapy
EGL: external germinal layer
PCL: Purkinje cell layer
PCs: Purkinje cells
GNs: granule neurons
RT: room temperature
TUNEL: terminal deoxynucleotidyl transferase-mediated deoxyuridine triphosphate nick end labeling
WT: wild-type
2-DE: two-dimensional electrophoresis
ROS: reactive oxygen species
Prdx2: peroxiredoxin 2
Prdx6: peroxiredoxin 6
Sod1: superoxide dismutase
Gnao: guanine nucleotide-binding protein G(o) subunit α
Eno2: enolase 2
