# Supplementary material for: Impairment of enzymatic antioxidant defenses is associated with bilirubin-induced neuronal cell death in the cerebellum of Ugt1 KO mice
Source: Cell Death Dis. 2015 May 7;6(5):e1739–. doi: 10.1038/cddis.2015.113 (PMC4669693; doi:10.1038/cddis.2015.113)
Supplement: Supplementary Information [file cddis2015113x1.doc]

**Impairment of enzymatic antioxidant defenses is associated with bilirubin-induced neuronal cell death in the cerebellum of Ugt1 KO mice**

Giulia Bortolussi^1*^, Erika Codarin^2*^, Giulia Antoniali^2^, Carlo Vascotto^2^, Simone Vodret^1^, Simona Arena^3^, Laura Cesaratto^2^, Andrea Scaloni^3^, Gianluca Tell^2#^ and Andrés F. Muro^1#^

*^1^International Centre for Genetic Engineering and Biotechnology, Trieste, Italy*

*^2^Department of Medical and Biological Sciences, University of Udine, 33100 Udine, Italy*

*^3^Proteomics & Mass Spectrometry Laboratory, ISPAAM, National Research Council, 80147 Naples, Italy*

**SupplementaRY material**

**Supplementary Methods**

**Evaluation of differentially represented spots**

Each sample was analyzed in quadruplicate and then, a comparative analysis was carried out. Protein spots were detected and matched between the different samples; individual spot volume values were obtained according to the program instructions. The staining intensities of matching spots between gels of the same sample were measured by the Image Master 2-D software (Amersham Biosciences, Milan, Italy). For each cropped image, the total volume of matched spots was set equal to 1.0 by using the program’s volume normalization function; then a normalized volume value was calculated for each matched spot. An analysis was performed on about 450 different matched spots from each sample. For each sample, the intra-experimental variability was evaluated by the linear regression analysis tool provided by the ImageMaster 2-D software. The different sample normalized volume values (relative intensity values) for the candidate proteins were visualized as histograms representing the ratios of the intensities of matched spots from WT vs MUT samples. Mean and median of logarithmic ratios of the intensity values for each pair of matched spots were determined. The latter values clustered around 0, as expected if the mean error of our analysis was normally distributed. The normal distribution was further demonstrated by the Kolmogorov – Smirnov test (performed by the Graph Pad computer software). Since the Kolmogorov – Smirnov parameter resulted close to 0, SDs of logarithmic ratio distributions were a valid parameter to assess the variability of our analysis. Then, for the differential display analysis we applied the *t*-Student test. In our experiments, the average value of logarithmic ratios of SD was 0.298 (for pH 4-7) and 0.391 (for pH 3-10) and therefore, a logarithmic ratio value above 0.596 (for pH 4-7) and 0.782 (for pH 3-10) was set as the cut-off point to evaluate protein species differentially expressed between distinct samples. From a statistical point of view, values over two SDs from the mean have less than 5% probability of representing not differentially expressed protein species and were considered as statistically significant with a *p*-value <0.05.

**Supplementary Table 1.** List of primers used in the Real Time PCR analysis.

| **Gene** | **Sequence 5’→3’** |
| --- | --- |
| mPCBP1DIR | AGCATCATCGGGAAGAAAGG |
| mPCBP1REV | GGCCTTAAAGATGGCATTGG |
| mPark7DIR | TACGATGTGGTGGTTCTTCC |
| mPark7 REV | AACCTACTTCGTGAGCCAAC |
| mDpysl3DIR | TCATCTCACAAGCCAGGAAG |
| mDpysl3REV | GCAAGGAGTTGATGTAGTCAGG |
| mEno2DIR | AGCAGGAGAAACTGGACAAC |
| mEno2REV | TGAGCAATGTGGCGATAGAG |
| mPrdx2DIR | ATATCCCTCTGCTTGCTGAC |
| mPrdx2REV | TTGACTGTGATCTGGCGAAG |
| mPrdx6DIR | TTGAGGATCATCTTGCCTGG |
| mPrdx6REV | TGTTGTTAGCGTCCTTCTCG |
| mSOD1DIR | GCGGTGAACCAGTTGTGTTG |
| mSOD1REV | CGGGCCACCATGTTTCTTAG |
| mYwhaeDIR | CAGCAGCATTGAACAGAAGG |
| mYwhaeREV | CAGTGTTAGCTGCTGGAATG |
| mNfe2l2DIR | GGCAGAGACATTCCCATTTG |
| mNfe2l2REV | AAACTTGCTCCATGTCCTGC |
| mGapdhDIR | ATGGTGAAGGTCGGTGTGAA |
| mGapdhREV | GTTGATGGCAACAATCTCCA |

**List of primers used in the Real Time PCR analysis.**

**Legend to Supplementary Table 2**. Distribution of the identified species according to Biological process and Molecular Function as obtained with GeneCodis analysis. ‘GO term ID’ and ‘Annotations’ columns represent the Gene Ontology codes of annotations and the textual description of annotations, respectively. Third and fourth columns represent the number of genes in the input list and the reference list for a given annotation, respectively. *p*-values calculated using hypergeometric distribution and its correction using the stimulation-based approach are reported. The ‘Genes’ column identifies the set of genes in the input list showing a given annotation.

| **GO term ID** | **Annotations** | **# of annotated genes in the input list (Total # of genes in the input list)** | **# of annotated genes in the reference list (Total # of genes in the reference list)** | **Hypergeometric Distribution (Fisher's exact test)** | **Corrected Hypergeometric Dist. (Fisher's exact test)** | **Genes** |
| --- | --- | --- | --- | --- | --- | --- |
| GO:0046872 | metal ion binding (MF) | 9(29) | 2802(37681) | 1.75E-04 | 3.49E-04 | Ndufv2,Tpm4,Lasp1,Gnao1,Fdps,Idh3a,Eno2,Sod1,Adh5 |
| GO:0000166 | nucleotide binding (MF) | 8(29) | 1999(37681) | 9.74E-05 | 2.60E-04 | Atp6v1a,Gnao1,Vat1,Sars,Psmc4,Pebp1,Psmc3,Adh5 |
| GO:0055114,GO:0016491 | oxidation-reduction process (BP),oxidoreductase activity (MF) | 7(29) | 603(37681) | 2.98E-07 | 2.39E-06 | Ndufv2,Prdx2,Idh3a,Prdx6,Vat1,Sod1,Adh5 |
| GO:0005515 | protein binding (MF) | 7(29) | 2999(37681) | 6.57E-03 | 9.28E-03 | Pcbp1,Prdx2,Lasp1,Ywhab,Apoa1,Sod1,Ywhae |
| GO:0016787 | hydrolase activity (MF) | 7(29) | 1501(37681) | 1.13E-04 | 2.72E-04 | Atp6v1a,Prdx6,Dpysl2,Psmc4,Park7,Psmc3,Dpysl3 |
| GO:0006979 | response to oxidative stress (BP) | 4(29) | 93(37681) | 7.88E-07 | 4.73E-06 | Prdx2,Prdx6,Park7,Sod1 |
| GO:0008270 | zinc ion binding (MF) | 4(29) | 1315(37681) | 1.75E-02 | 2.10E-02 | Lasp1,Vat1,Sod1,Adh5 |
| GO:0008270,GO:0055114,GO:0016491 | zinc ion binding (MF),oxidation-reduction process (BP),oxidoreductase activity (MF) | 3(29) | 40(37681) | 3.97E-06 | 1.59E-05 | Vat1,Sod1,Adh5 |
| GO:0051219 | phosphoprotein binding (MF) | 3(29) | 39(37681) | 3.68E-06 | 1.76E-05 | Ywhab,Dpysl3,Ywhae |
| GO:0055114,GO:0016491,GO:0016209,GO:0006979 | oxidation-reduction process (BP),oxidoreductase activity (MF),antioxidant activity (MF),response to oxidative stress (BP) | 3(29) | 7(37681) | 1.43E-08 | 1.72E-07 | Prdx2,Prdx6,Sod1 |
| GO:0042803 | protein homodimerization activity (MF) | 3(29) | 527(37681) | 7.59E-03 | 1.01E-02 | Eno2,Park7,Adh5 |
| GO:0007399 | nervous system development (BP) | 3(29) | 279(37681) | 1.27E-03 | 2.04E-03 | Ndufv2,Dpysl2,Dpysl3 |

**Supplemental Table 3.** List of the antibodies used for Western blot (WB) and immunofluorescence (IF) analyses.

|  | Protein | Code | Company | Working Dilution |
| --- | --- | --- | --- | --- |
| **1** | Caspase 3 total | sc-7148 | Santa Cruz | 1:500 (WB) |
| **2** | Cleaved caspase 3 | 9661 | Cell Signaling | 1:800 (WB) |
| **3** | CalbininD28K | 214011  214002 | SYSY  SYSY | 1:3000 (WB); 1:400 (IF)  1:3000; 1:400 (IF) |
| **4** | DJ1 | sc-27006 | Santa Cruz | 1:500 (WB); 1:100 (IF) |
| **5** | Dpysl3 | ab-126787 | Abcam | 1:2000 (WB); 1:100 (IF) |
| **6** | Eno2 | ab-53025 | Abcam | 1:1000 (WB); 1:100 (IF) |
| **7** | NeuN | ABN78 | Cell Signaling | 1:100 (IF) |
| **8** | PCBP1 | ab-74793 | Abcam | 1:1000 (WB); 1:100 (IF) |
| **9** | Prdx2 | ab-109367 | Abcam | 1:1000 (WB); 1:100 (IF) |
| **10** | Prdx6 | ab-92322 | Abcam | 1:1000 (WB); 1:100 (IF) |
| **11** | P-P38 | 4511 | Cell Signaling | 1:1000 (WB); 1:100 (IF) |
| **12** | P38 | 9212 | Cell Signaling | 1:1000 (WB); 1:100 (IF) |
| **13** | Sod1 | ab-16831 | Abcam | 1:1000 (WB); 1:100 (IF) |
| **14** | 14-3-3e | ab-40117 | Abcam | 1:1000 (WB); 1:100 (IF) |
| **15** | Tubulin | E7 | Developmental Studies Hybridoma Bank | 1:5000 (WB) |

**List of antibodies used in Western blot (WB) and Immunofluorescence (IF) analyses.**

**Legends to the Supplementary Figures**

**Supplementary Figure 1. SDS-PAGE profiles of cerebellar proteins.**

12 μg of total protein extracts obtained by processing cerebella from Ugt1 mutant and WT 4-days-old mice were separated onto 10% SDS-PAGE and stained with colloidal Coomassie blue. SDS-PAGE profiles were comparable for all the analyzed samples without any trace of protein degradation, confirming the efficacy of the protein extraction protocol adopted. R represents the reference gold standard sample of protein extraction protocol.

**Supplementary Figure 2. 2-DE analysis of cerebellar proteins.**

Proteins over- or down-represented in mutant mice cerebella. Representative gel regions comprising some of the statistically significant changes in the proteome repertoire were cropped. In the figure presented are only the protein spots not displayed in the main manuscript.

**Supplementary Figure 3. 2-DE Western blot analysis of cerebellar proteins.** Protein extracts from Ugt1 mutant and WT mice cerebella were loaded onto 7 cm, pH 3-10 L (35 µg of protein extracts) and pH 4-7 (70 µg of protein extracts) IPG strips and then electrophoresed onto 12% SDS-PAGE. Proteins were transferred to nitrocellulose membranes and then incubated with specific antibodies. Western blotting was performed to detect Pcbp1 (Panel A), DJ-1 (Panel B), 14-3-3e (Panel C), Eno2 (Panel D), Prdx2 (Panel E) and Prdx6 (Panel F) protein representation. Actin was used as loading control. Densitometric values, expressed as percentage, were reported as histograms of the ratio between the analyzed protein and actin band intensities. For Dpysl3, the quantification of the single spot identified is reported and expressed as fold change relative to WT mice. Western blot validation data are the mean of two independent experiments on separate biological replicas. In each case, variation was less than 15%.

**Supplementary Figure 4. Evaluation of the extent of inter-individual variability for the differentially represented protein species in each group.** Western blot analysis performed on 30 µg of total cerebellar protein samples used for 2-DE analysis reported in Figure 2. WT and Ugt1 mutant samples analyzed were obtained from the mice specimens (n=4) used for the 2-DE analysis plus two new ones. Samples were separated onto 12% SDS-PAGE. In order to evaluate inter-individual variability in the same group (intra-group), WT and mutant samples groups were run into separate gels. A representative image of Western blot analysis obtained for Dpysl3, Eno2, Pcbp1, 14-3-3e, Sod1 and DJ-1 is shown. Actin was used as loading control and for the relative normalization. To evaluate inter-group inter-variability in the expression fold of each protein among WT and mutant samples, band intensities were acquired and quantified within the same scan area. Plots indicate the different distributions of the fold of protein expression, normalized for actin content, for all the proteins. Each circle represents the mean of three Western blot replicates. Mean and standard deviation of the distribution are indicated. Errors bars SD. *t*-test * *p*< 0.05.
