# Supplementary figures and images for: Impairment of enzymatic antioxidant defenses is associated with bilirubin-induced neuronal cell death in the cerebellum of Ugt1 KO mice
Source: Cell Death Dis. 2015 May 7;6(5):e1739–. doi: 10.1038/cddis.2015.113 (PMC4669693; doi:10.1038/cddis.2015.113)

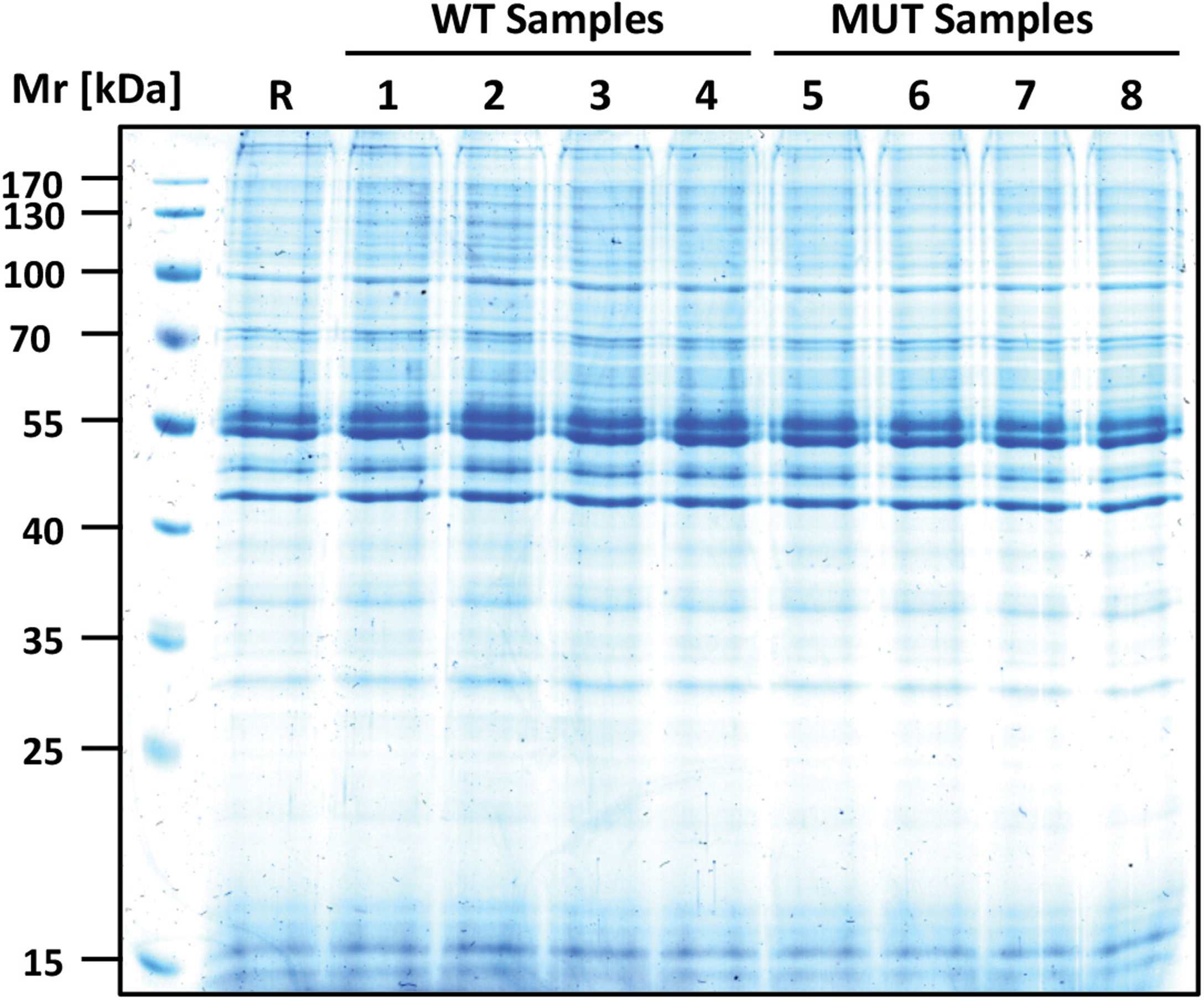

Supplement: Supplementary Figure 1 [file cddis2015113x2.tif]

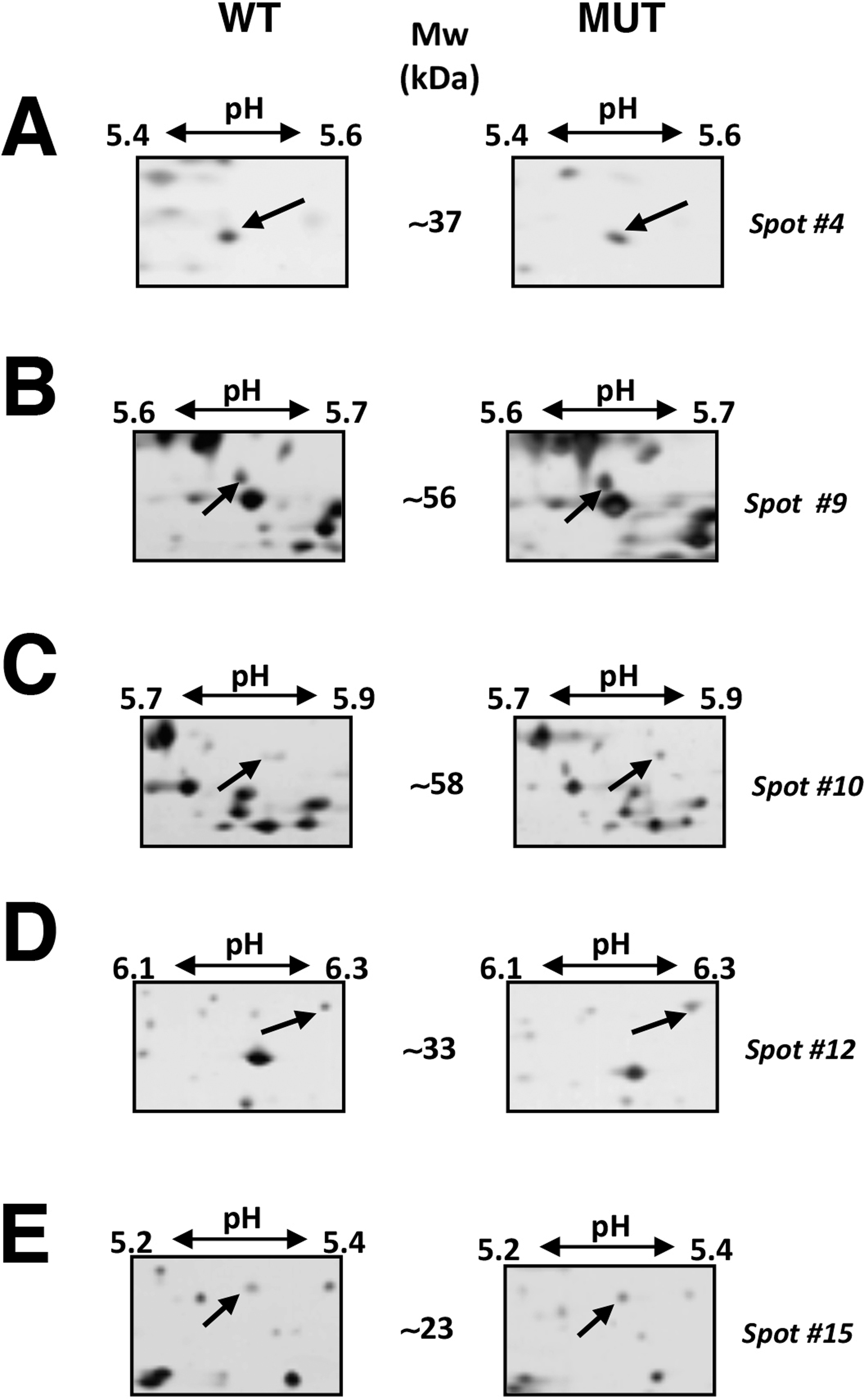

Supplement: Supplementary Figure 2 [file cddis2015113x3.tif]

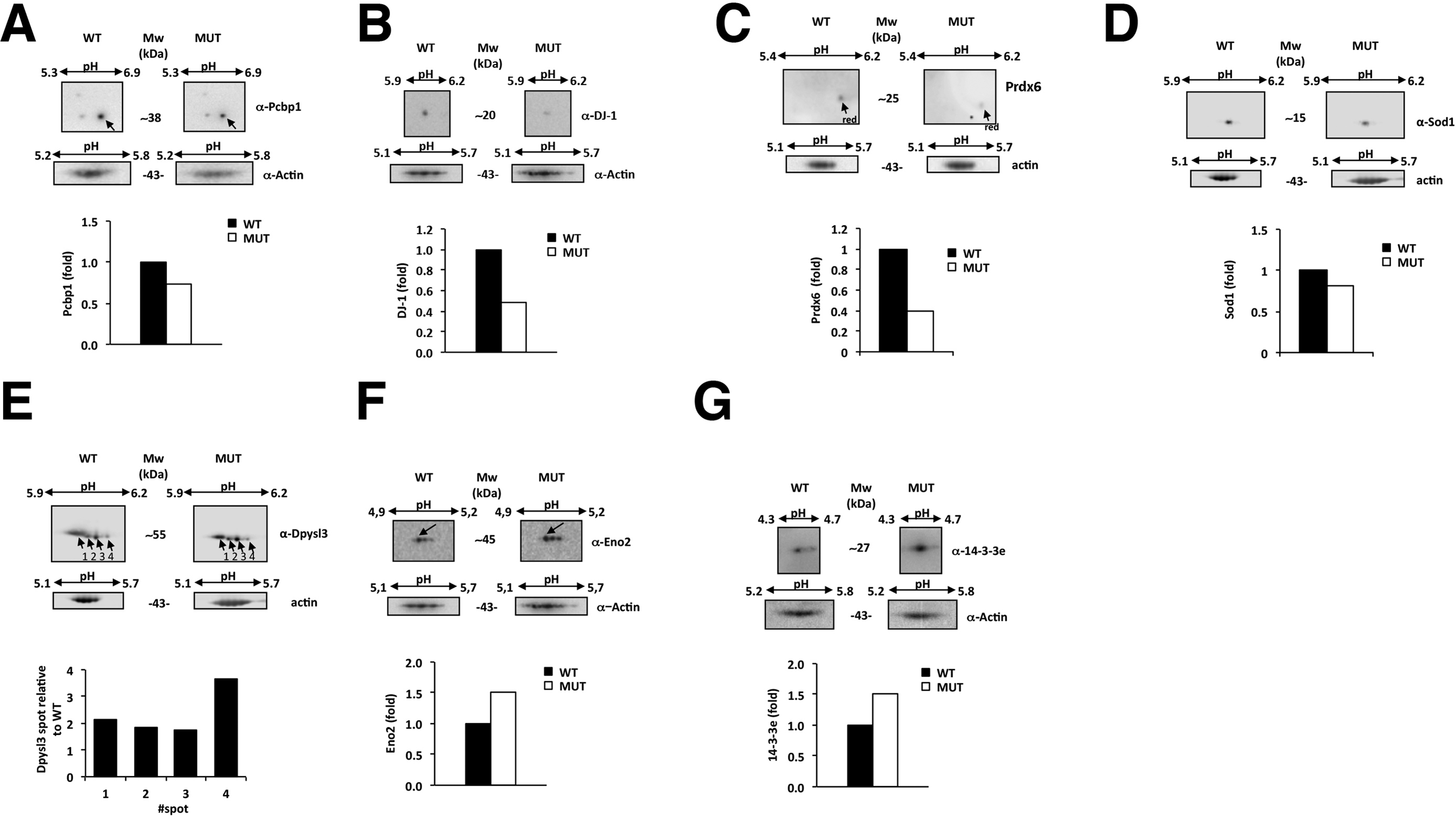

Supplement: Supplementary Figure 3 [file cddis2015113x4.tif]

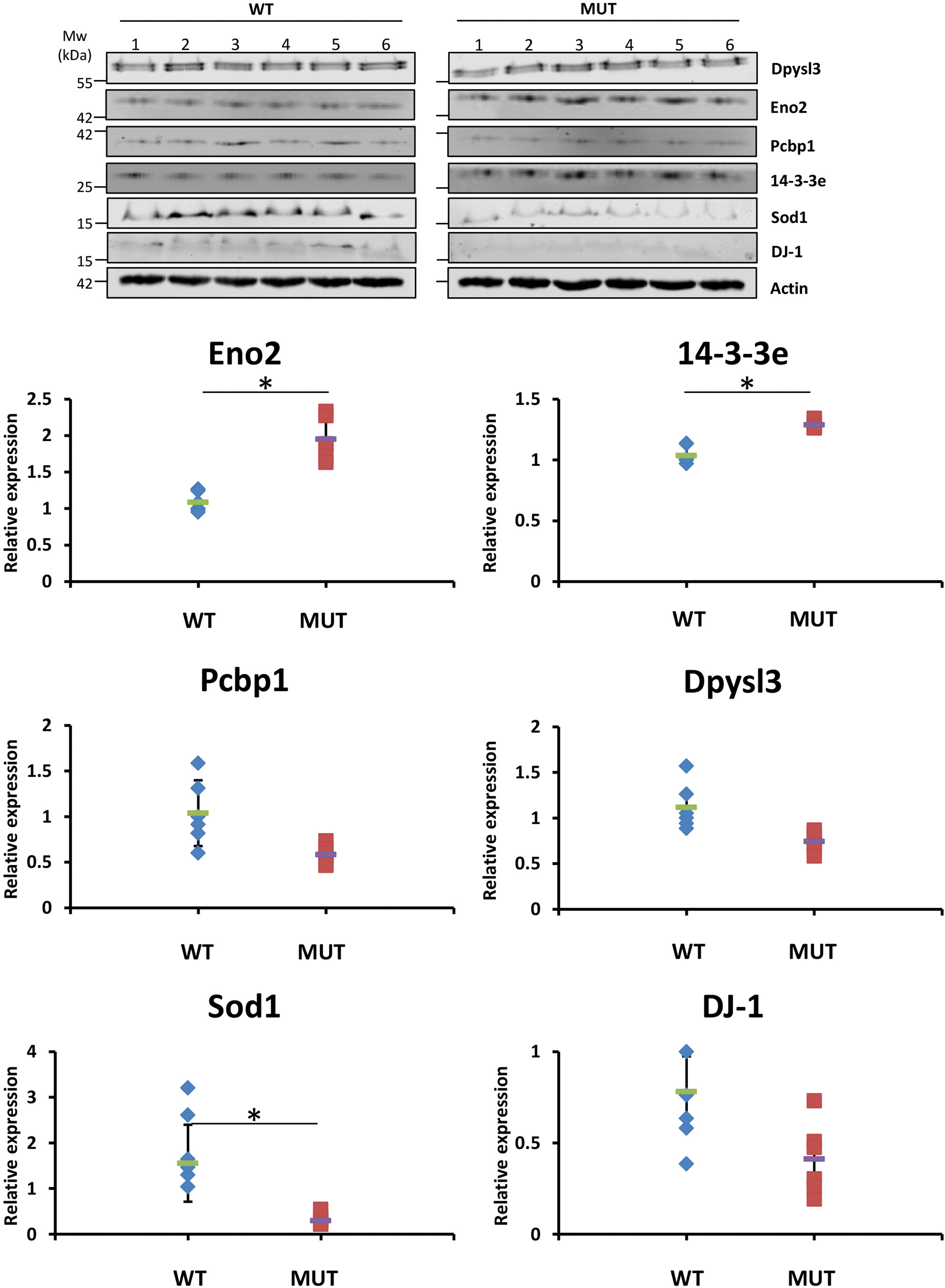

Supplement: Supplementary Figure 4 [file cddis2015113x5.tif]
